# Undiagnosed Diabetes and Pre-Diabetes in Health Disparities

**DOI:** 10.1371/journal.pone.0133135

**Published:** 2015-07-17

**Authors:** Susan P. Fisher-Hoch, Kristina P. Vatcheva, Mohammad H. Rahbar, Joseph B. McCormick

**Affiliations:** 1 Division of Epidemiology, University of Texas Health Science Center, Houston, School of Public Health, Brownsville Campus, Brownsville, Texas, United States of America; 2 Division of Epidemiology, Human Genetics and Environmental Sciences, University of Texas School of Public Health, Division of Clinical and Translational Sciences, Department of Internal Medicine, University of Texas Medical School at Houston, and Center for Clinical and Translational Sciences at The University of Texas Health Science Center at Houston, Room 1100.05 UT Professional Building, Houston, Texas, United States of America; Medical University Innsbruck, AUSTRIA

## Abstract

Globally half of all diabetes mellitus is undiagnosed. We sought to determine the extent and characteristics of undiagnosed type 2 diabetes mellitus and pre-diabetes in Mexican Americans residing in the United States. This disadvantaged population with 50% lifetime risk of diabetes is a microcosm of the current pandemic. We accessed baseline data between 2004 and 2014 from 2,838 adults recruited to our Cameron County Hispanic Cohort (CCHC); a two-stage randomly selected ‘Framingham-like’ cohort of Mexican Americans on the US Mexico border with severe health disparities. We examined prevalence, risk factors and metabolic health in diagnosed and undiagnosed diabetes and pre-diabetes. Two thirds of this Mexican American population has diabetes or pre-diabetes. Diabetes prevalence was 28.0%, nearly half undiagnosed, and pre-diabetes 31.6%. Mean BMI among those with diabetes was 33.5 kg/m^2^ compared with 29.0 kg/m^2^ for those without diabetes. Significant risk factors were low income and educational levels. Most with diabetes had increased waist/hip ratio. Lack of insurance and access to health services played a decisive role in failure to have diabetes diagnosed. Participants with undiagnosed diabetes and pre-diabetes had similar measures of poor metabolic health similar but generally not as severe as those with diagnosed diabetes. More than 50% of a minority Mexican American population in South Texas has diabetes or pre-diabetes and is metabolically unhealthy. Only a third of diabetes cases were diagnosed. Sustained efforts are imperative to identify, diagnose and treat individuals in underserved communities.

## Introduction

In the United States (U.S.) rates of diagnosed type 2 diabetes mellitus have increased from 6.2% (1988–1994) to 10.2% (2005–2010). The overall lifetime risk of diabetes (2000–2011) is 40.2% for men and 39.6% for women.[1,2] The lifetime diabetes risk in minorities, however, is markedly higher; 44.7% and 55.3% for African American men and women respectively, and 51.8% and 51.5% for Hispanic men and women. Hidden within these estimates are large numbers of undiagnosed diabetes, particularly in disadvantaged populations.[2] Though the proportion of undiagnosed diabetes in the United States fell from 2005–2010, this was mainly accounted for by whites. In minorities rates of undiagnosed diabetes rose; in Mexican Americans from 1.8% to 2.9% and in African Americans from 1.8 to 2.4%. Overall diabetes in people of Mexican American origin rose from 5.5% to 8.5%.[2] In minority populations many of those who are diagnosed fail to take diabetes medication.[3] By definition the undiagnosed are untreated. There is therefore a large pool of people who need but do not access diabetes treatment, particularly in concentrated areas of extreme health disparity where the burden of disease is usually underestimated.[4]

The burden of diabetes is a global problem, and the United States is not spared. Most people with diabetes are between the ages of 40 and 59 years and 80% live in low- and middle-income countries (LMICs).[5] Nearly half of diabetes cases globally are undiagnosed.[5] In the United States minority communities where conditions are similar to those in LMICs also have high rates of undiagnosed diabetes, particularly in minority groups, notably Hispanics.[4,6–8] By 2050 Hispanics will comprise 30% of the population, among whom the largest sub-population [58.5%] is Mexican-American with the highest lifetime risk of diabetes (over 50%).[2,9,10] Hispanics, mostly of Mexican origin, comprise 40% of the population of Texas counties.[11] On the Mexico border in Cameron County as well as the rest of the Lower Rio Grande Valley, 88% or more of the population is of Mexican origin. Here the overall prevalence of diagnosed diabetes at 15.7% is much higher than the national average of 8.3%, and diabetes was the sixth leading cause of death responsible for 14,513 deaths in 2003.[12] The data and the shift in demographics presage the urgent need for increased attention to the health burdens of diabetes, diagnosed and undiagnosed, and pre-diabetes in disadvantaged populations.

We have developed a community-based Mexican American cohort in Cameron County, south Texas, on the Texas/Mexico border, with health care insurance rates of only 30%. Under-sampling of minority populations with health disparities such as ours likely results in underestimates of prevalence. Overall reported prevalence of diabetes, diagnosed and undiagnosed, in Mexican Americans in the lower Rio Grande Valley, and along the U.S. Mexico Border, and diabetes-specific mortality, has been historically higher in south Texas counties than elsewhere.[4,7,8] Known risk factors associated with developing diabetes include race/ethnicity, older age, less education, lack of insurance, obesity, family, and smoking history.[13] All of these risk factors are prominent in the population of Cameron County, as they are along the entire Texas/Mexico border. Our objective in this report is to determine the extent and characteristics of undiagnosed diabetes and pre-diabetes in Mexican Americans in order to draw attention to populations in the U.S. and elsewhere with high rates of undiagnosed diabetes presaging considerable social and economic cost.

## Methods

The Committee for the Protection of Human Subjects at the University of Texas Health Science Center at Houston approved the study protocol, written consent forms and procedures and free and informed consent was obtained from all subjects. The investigators had no conflict of interest to disclose at consent.

### Study population

This is a cross-sectional study using baseline data from 2,856 adults recruited to the CCHC between 2004 and 2014, aged 18 years and older. The population sampled is from one of the two poorest counties by size in the United States. Participants were recruited in Brownsville, Texas, through two-stage cluster sampling of households from randomly selected census tracts using Census 2000.[8]

### Participant Recruitment

Members from randomly selected households were invited to visit our Clinical Research Unit (CRU). After obtaining written informed consent, we administered questionnaires assessing socio-demographic and anthropomorphic characteristics, clinical examinations, specimen collection, and clinical laboratory estimations as previously described.[8] Anthropomorphic biomarkers of poor metabolic health, such as waist-to-hip ratio were used as a surrogate marker of abdominal adiposity.[14] English and Spanish literacy scores were obtained using the Mini-Mental State Examination protocols (Psychological Assessment Resources, Inc., Lutz, Florida).[15]

## Definitions

Participants were considered obese if their Body Mass Index (BMI) was ≥ 30 kg/m^2^. We used the 2010 American Diabetes Association (ADA) definition of diabetes throughout. This is based on fasting glucose or oral glucose tolerance test (OGTT) and/or classical symptoms, to which glycated hemoglobin (HbA1c) as a single diagnostic criterion was added in 2010.[16] If a healthcare provider had told a participant they had diabetes or if a participant was on hypoglycemic medications, they were considered to have self-reported (previously diagnosed) diabetes. Participants were considered to have undiagnosed diabetes if they responded ‘no’ to these questions but otherwise met the ADA 2010 definition for diabetes.[16]

### Laboratory Methods

Fasting blood specimens were collected, processed and stored as previously described.[8] Specimens were sent to a Clinical Laboratory Improvement Amendments-approved (CLIA) laboratory for measurement of clinical chemistries and other blood estimations. The range of biomarkers used were those indicative of poor metabolic health. A Glucostat analyzer (Model 27, YSA Inc. Yellow Springs, OH) was used to determine fasting glucose. Insulin levels were determined using ELISA assays (Mercodia, Uppsala, Sweden). Insulin resistance was determined using the Homeostasis Model Assessment (HOMA-IR = glucose (mg/dL)/18 x insulin (mU/L)/22.5) equation.[17]

### Statistical Analysis

Descriptive statistics using sampling weights provide information regarding the population. Data stratified by diabetes status are summarized as weighted mean and standard error for continuous variables and frequency and weighted percentages for categorical variables. SI units are used throughout. The sampling weights were created to account for imbalances in the distribution of sex and age due to unequal participation of household members in the census tracts sampled.[8] A multivariable, weighted logistic regression model was built to determine independent factors influencing diabetes status. The model assumptions were tested and categorization of independent variables was performed. The main effects of all selected variables and interactions between them were examined using the Wald test at significance levels of 0.05. The model is tested for goodness of fit using Archer and Lemeshow’s design–adjusted test at 0.05 level of significance. The analyses were performed using SAS 9.2 TS level 1MO (SAS Institute Inc., Cary, NC) and STATA/IC 12.1 (StataCorp LP, College Station, TX).

### Role of the funding sources

The funding sources had no role in the study design, collection, analysis, interpretation of data, writing of the report, or decision for submission. SF-H has access to all data in the study and final responsibility for publication.

## Results

The weighted prevalence of diabetes measured in this community was 27.6%; that is, nearly one in three of all adults aged 18 years and older have diabetes (Table 1). (This compares with 20.6% prevalence using the 2006 ADA definition which does not include HbA1c.) Forty percent of those with diabetes (345 participants) were previously undiagnosed. An additional 32.0% had pre-diabetes. The profile of participants with diabetes whether diagnosed or undiagnosed is similar. Overall participants with diabetes reported two years less education (95% CI -2.90, -1.07, p-value<0.0001) and lower annual income (mean difference-$5,570, 95% CI-$13,483, $2,341, p-value = 0.0111) than those without diabetes. Compared with those without, those with diabetes are more likely to be obese, older, with low income and poor education.

**Table 1:** Weighted prevalence and means of key socio-demographic and anthropomorphic characteristics of the CCHC population. Prevalence is reported using weighted percentages.

Categorical variables	Total (n = 2838) n (%)	Diabetes (n = 795) n (%)	No diabetes (n = 2043) n (%)	Odds ratio for diabetes (95% CI)	p value
Diabetes	795 (27.6)				
Diagnosed diabetes*	449 (16.4)	449 (60.0)			
Undiagnosed diabetes	345 (11.2)	345 (40.0)			
Pre-diabetes	889 (32.0)				
Obese	1456 (50.9)	507 (65.7)	949 (45.3)	1.95 (1.43, 2.67)	<0.001
Overweight	910 (33.3)	205 (26.6)	705 (35.8)	Reference	
Males	970 (43.4)	279 (43.5)	691 (43.4)	1.01 (0.77, 1.31)	0.9679
Females	1868 (56.6)	516 (56.5)	1352 (56.6)	Reference	
Born in Mexico	1817(59.6)	534 (62.0)	1283 (58.6)	1.15 (0.89, 1.49)	0.2949
Born in U.S.	976 (40.4)	252 (38.0)	724 (41.4)	Reference	
With health insurance	833 (34.9)	536 (41.8)	297 (32.3)	1.50 (1.13, 2.00)	0.0072
Heavy smoker**	863 (33.7)	260 (38.2)	603 (32.0)	1.32 (1.00, 1.73)	0.0486
Alcohol User	1409 (52.8)	367 (49.2)	1042 (54.1)	0.82 (0.64, 1.05)	0.1178
Continuous variables	Mean (SE)	Mean (SE)	Mean (SE)	Mean difference (95% CI)	p value
Age in years	46.0 (0.68)	53.8 (1.14)	43.1 (0.74)	10.6 (8.12, 13.07)	<0.0001
Annual income	$23, 561 ($1, 019)	$20, 178 ($1, 334)	$24, 950 ($1, 319)	-$4, 771(-$8, 454,-$1,089)	0.0111
Years education	12.9 (0.12)	11.9 (0.25)	13.3 (0.13)	-1.46 (-2.00, -0.92)	<0.0001
Spanish test score	37.8 (0.32)	35.4 (0.71)	38.6 (0.34)	-3.23 (-4.78, -1.70)	<0.0001
English test score	31.0 (0.50)	27.2 (1.02)	32.3 (0.57)	-5.18 (-7.45, -2.90)	<0.0001
Years resident in Brownsville	23.70 (0.76)	29.2 (1.46)	21.6 (0.69)	7.55 (4.92, 10.18)	<0.0001

*A participant who has been told by a doctor they have diabetes

**(∠100 cigarettes/day)

Table 1 also shows participants with diabetes who had health insurance were more likely to have been diagnosed and therefore self-report diabetes than those who were uninsured (OR 1.97, 95% CI 2.25, 3.10, p-value = 0.0072). Participants with diagnosed diabetes were on average 8.7 years older (95%CI 4.72, 12.63, p-value<0.0001) than the undiagnosed, due in part to the high proportion of older diagnosed individuals receiving Medicare (health insurance for Americans aged 65 and older who have worked and contributed into the system).

When compared with participants with diabetes with no insurance, participants with diabetes on Medicaid were significantly more likely to be diagnosed (OR 4.45, 95%CI 2.18, 9.08, p-value<0.0001) as were those with Medicare (OR 2.24, 95%CI 1.01, 4.96, p-value = 0.0140), but in this community, those with private insurance were no more likely to be diagnosed than those without insurance (OR 0.75, 95%CI 0.35–1.61, p-value = 0.5220). (Medicaid provides free health care for families and individuals with the lowest incomes and limited resources.)

Table 2 shows the association with diabetes of adverse metabolic markers for chronic diseases. The anticipated association of obesity with diabetes was particularly marked in the morbidly obese and those with higher waist-to-hip ratios (85.6% of participants with diabetes had elevated waist-to-hip ratios). Mean HOMA-IR levels were nearly three times above the upper limits of normal in diabetes. Participants with diabetes also had higher blood pressures, triglycerides, and C-reactive protein (CRP) levels than those without diabetes. We further examined these findings in a multivariable model. Here we found that elevated C-reactive protein, and older age remained independently associated with diabetes in addition to abnormal waist-to-hip ratios. There was a significant interaction between elevated HOMA-IR and elevated waist-to-hip ratios associated with diabetes indicating that the effect of HOMA-IR on diabetes depended on the value of waist-to-hip ratio. We found that the odds ratio for abnormal HOMA-IR in diabetes is 3.48 (95% CI 2.54, 4.76, p-value<0.0001) for individuals with abnormal waist-to-hip ratio. The odds ratio for abnormal HOMA-IR in diabetes for individuals with normal waist-to-hip ratio was not statistically significant. When examining only those participants with diabetes who had an abnormal HOMA-IR and controlling for all other significant variables, we found that the odds ratio for abnormal waist-to-hip ratio in diabetes was 3.01 (95%CI 1.79, 5.06, p-value<0.0001).

**Table 2:** Comparison of weighted means of key metabolic and biomarker characteristics (SI units) of the CCHC population, between participants who fulfill the 2010 American Diabetes Association definition of diabetes and those who do not, excluding pre-diabetes. (N = 1949).

	Diabetes (n = 795) Mean (SE)	No Diabetes* (n = 1154) Mean (SE)	Mean difference (95%CI)	p values
Waist-to-hip ratio	0.96 (0.004)	0.91 (0.003)	0.05 (0.04, 0.06)	<0.0001
BMI (kg/m^2^)	33.5 (0.44)	29.0 (0.26)	4.48 (3.50, 5.45)	<0.0001
Insulin (pmol/l)	105.2 (3.86)	77.8 (2.82)	27.34 (17.89, 36.79)	<0.0001
Insulin resistance (HOMA)	40.0 (1.71)	17.4 (0.64)	22.69 (1.09, 26.28)	<0.0001
Glycated hemoglobin (HbA1c) %	7.8 (0.14)	5.0 (0.03)	2.8 (2.47, 3.03)	<0.0001
mmol/mol IFCC /%	62 (1.5)	28 (0.4)	10 (9, 13)	<0.0001
Diastolic blood pressure (mmHg)	71.5 (0.56)	69.8 (0.45)	1.66 (0.27, 3.06)	0.0201
Systolic blood pressure (mmHg)	122.2 (0.92)	111.9 (0.66)	10.35 (8.15, 12.54)	<0.0001
C-reactive protein (nmol/L)	87.5 (7.30)	64.6 (5.5)	22.91 (5.54, 4.27)	0.0097
Triglycerides(mmol/L)	2.3 (0.10)	1.5 (0.04)	0.79 (0.58, 1.01)	<0.0001
Total cholesterol (mmol/L)	4.7 (0.06)	4.7 (0.04)	0.01 (-0.13, 0.14)	0.9331
High density lipoprotein (mmol/L)	1.2 (0.02)	1.3 (0.02)	-0.09 (-0.13, -0.05)	<0.0001
Low density lipoprotein (mmol/L)	2.8 (0.05)	2.8 (0.04)	-0.14 (-0.26, -0.01)	0.0308
Alanine aminotransferase (AST) (U/L)	0.7 (0.02)	0.7 (0.02)	0.06 (0.003, 0.13)	0.0376
Aspartate aminotransferase (ALT) (U/L)	0.6 (0.02)	0.6 (0.02)	0.003 (-0.05, 0.06)	0.8965

*excluding pre-diabetes

## Discussion

Two thirds of the minority Mexican American population we studied had diabetes or pre-diabetes and all had poor metabolic health. Diagnosed diabetes has been described as the tip of the iceberg.[18] Indeed, in our study, only just over half of those with diabetes were previously diagnosed. Inequity such as we describe, in the U.S. health care system has been described as ‘troubling’.[19] The impact of this Health disparity in a minority community in the United States differs little from the global inequality in chronic disease care in LMICs.[20] Failure to address undiagnosed and pre-diabetes is a major threat to health and economies.

We make three substantial observations. Firstly, nearly one third of a Mexican American population in the US has diabetes, and another third pre-diabetes. This is a tremendous pool of high risk people for a plethora of serious complications with daunting long term health consequences. Secondly, lack of health insurance and access to health services plays a decisive role in lack of diagnosis and treatment of diabetes. Even then we have shown that many in this cohort who are diagnosed are without treatment.[4] Thirdly, the similar metabolic profiles of diagnosed and undiagnosed diabetes demonstrates the urgency of widespread screening. This includes early detection of pre-diabetes where intervention can be most effective. Regardless, up to two thirds of this and many other populations already have diabetes, or are at imminent risk. We conclude that a much more aggressive and broader screening campaign must be mounted if we are to lower the inevitable burden of disease for the future.

Lack of health insurance is a major barrier to diagnosis of diabetes, but we find Medicaid provides the best access to diabetes diagnosis.[21] Having Medicare also increases the likelihood of diagnosing diabetes compared to private insurance. The failure of private health insurance to equal Medicaid and Medicare rates of diagnosis is disturbing and requires further study, and suggests there is more to diabetes diagnosis than simple access to care. Perhaps the stringent rules of Medicaid and Medicare ultimately facilitate better, and more uniform care than those under private insurance. It is also possible that denial and avoidance play an important role. Another issue shown in Table 1 is that the odds ratio for having diabetes in those with insurance is 1.5 time those without. This can be explained by the fact that more than a third of the insured have Medicare who are older and therefore most likely to have diabetes. Another third of the insured are on Medicaid. The numbers with private insurance are very small, so the significance of rates in these people is unclear. [4]

In this study we clearly show the close association with increasing BMI. However as others have shown waist-to-hip ratio appears to be a more specific factor identifying those with, or at risk of diabetes.[13] This ratio is an indirect measurement of the amount of metabolically active visceral fat, and is known to be associated with other chronic diseases, particularly cardiovascular disease.[22] Using this approach, Tables 2 and 3 suggest the most specific, but obvious and inexpensive profile for primary screening for undiagnosed diabetes at the population level is high waist-to-hip ratio. This is supported by the multivariable model which shows that an abnormal HOMA-IR along with older age, and waist-to-hip ratio are associated with type 2 diabetes (Table 3). Waist-to-hip ratio is already established as an increased risk for myocardial infarction, stroke and premature death and is clearly also a risk for diabetes.[23]

**Table 3:** Comparison of unadjusted and age-adjusted weighted mean metabolic and biomarker characteristics in CCHC participants between diagnosed and undiagnosed diabetes, (n = 1683)

	Pre-diabetes (n = 889) Mean (SE)	Diagnosed Diabetes (n = 449) Mean (SE)	Undiagnosed Diabetes (n = 345) Mean (SE)	Mean difference Diagnosed vs undiagnosed (95%CI)	p value
Waist-to-hip ratio	0.94 (0.004)	0.97 (0.01)	0.94 (0.01)	0.03 (0.02, 0.05)	<0.0001
BMI kg/m^2^	31.5 (0.34)	33.6 (0.6)	33.3 (0.61)	0.28 (-1.39, 1.94)	0.7498
Insulin (pmol/l)	107.6 (3.62)	98.3 (4.1)	115.5 (7.25)	-17.27 (-33.55, -1)	0.0375
Insulin resistance (HOMA)	27.2 (0.97)	40.3 (1.99)	39.6 (3.07)	0.73 (-6.44, 7.89)	0.8494
Glycated hemoglobin (HbA1c) %	5.4 (0.1)	7.7 (0.2)	7.8 (0.2)	-0.9 (-7.0, 5.2)	0.7662
mmol/mol [IFCC]/%	36 (0.5)	61 (2.0)	62 (2.4)	-0.1 (-0.6, 0.5)	0.7662
Mean fasting blood glucose (nmol/L)	5.6 (0.02)	9.4 (0.23)	7.5 (0.3)	1.9 (1.16, 2.64)	<0.0001
Diastolic blood pressure	72.6 (0.62)	69.5 (0.7)	74.4 (0.89)	-4.87 (-7.11, -2.62)	<0.0001
Systolic blood pressure	118.7 (1.07)	123.2 (1.24)	120.8 (1.36)	2.43 (-1.21, 6.08)	0.1845
C-reactive protein (nmol/L)	105.9 (12.42)	94 (9.84)	78 (10.69)	15.95 (-12.41, 44.32)	0.2726
Triglycerides (mmol/L)	1.9 (0.07)	2.4 (0.13)	2.1 (0.15)	0.25 (-0.13, 0.64)	0.1965
Total cholesterol (mmol/L)	4.8 (0.05)	4.7 (0.07)	4.7 (0.08)	-0.03 (-0.24, 0.19)	0.7984
High density lipoprotein (mmol/)	1.2 (0.02)	1.2 (0.02)	1.2 (0.03)	-0.03 (-0.09, 0.04)	0.4625
Low density lipoprotein (mmol/L)	2.9 (0.05)	2.6 (0.06)	2.7 (0.08)	-0.13 (-0.33, 0.06)	0.1840
Alanine aminotransferase (U/L)	0.7 (0.02)	0.7 (0.03)	0.8 (0.03)	-0.11 (-0.2, -0.02)	0.0194
Aspartate aminotransferase (U/L)	0.6 (0.02)	0.6 (0.03)	0.6 (0.05)	-0.07 (-0.17, 0.03)	0.1748

Insulin resistance has been well documented as a strong predictor for the development of type 2 diabetes.[24–26] In this population it is associated with Amerindian ancestry, perhaps also an important contributor to the high prevalence of diabetes.[27] Participants with diabetes were also more likely to have abnormal levels of the biomarker of chronic inflammation, CRP, when compared to participants without diabetes. Elevated CRP is frequently reported in association with diabetes and has been shown to predict all-cause mortality in European origin Americans with type 2 diabetes.[28] Over 60% of CCHC participants with diabetes have significantly elevated CRP levels. Of additional interest, participants in the CCHC have previously been reported to have high prevalence of biomarkers of non-alcoholic fatty liver disease (NAFLD).[29–31] We confirm the association of elevated liver enzymes and diabetes in this study. The elevated liver enzymes add the specter of chronic liver disease to the multitude of ailments and health disparities awaiting undiagnosed diabetes.[32] The potentially devastating effects of NAFLD (cirrhosis and liver cancer) are, like obesity and diabetes, most amenable to prevention at early stages. Successful prevention of diabetes would reduce the burden of many important chronic diseases from cardiovascular to renal and liver disease to dementia and certain cancers.[33]

Those who are already diagnosed with diabetes appear to have evidence of more visceral fat, with more marked adverse metabolic profiles (raised triglycerides), higher frequency of abnormal insulin metabolism and higher fasting blood glucose than those who are undiagnosed. It may be that increasing burden of symptoms led these people to seek medical care, and thus diagnosis. There is a trend for diagnosed participants to have higher glycated hemoglobin levels (HbA_1C_) but this is not statistically significant. An issue related to lack of diagnosis we also observe in this population is a reluctance to confront the diagnosis and thus the consequences. This may in part be due to lack of insurance, and thus access to care, but the general reluctance to face additional life challenges is key, particularly among the disadvantaged.[21]

This study had several limitations. We did not use the oral glucose tolerance test (OGTT) because of participant time and cost constraints, but had we done so, the measured prevalence of abnormal glucose metabolism might have been higher, and the associations tighter since we would have removed participants with missed diabetes from the control group. In any event, diagnosis by fasting blood sugar, glycated hemoglobin or OGTT are not always concordant.[16] By using only diabetes prevalence and risk factor data from a single city in Texas, the results may not be representative of Hispanics in the entire region or state. Nevertheless, a great strength of this unique study was the use of a culturally and genetically homogenous population-based, under sampled, minority cohort to develop extensive and detailed data. The design also avoids biases from clinic sampled populations, or national data which have low sampling frequency in pockets of minorities and the uninsured such as this one. These populations rarely have landlines, so telephone surveys miss significant pathology. The cross-sectional study design also lacks the strength of longitudinal outcome data, but these studies are underway since the CCHC is designed as a longitudinal study to generate outcome data as well as to implement and evaluate interventions. By capturing data in the community before serious medical events supervene, we open doors for both intervention and prevention.

Two critical questions remain for our future. How do we find and treat the large number of people with undiagnosed diabetes, and how do we prevent those with pre-diabetes from developing diabetes on a large scale? These people are the hardest to identify in disadvantaged populations. The challenge is to detect diabetes early in the disease course when prevention and intervention are more easily achieved.[34]

## Conclusions

In summary, these data also sound the urgent alarm for the need to implement new evidence-based prevention and intervention strategies which involve the communities themselves.[35] The task is pressing. Abdominal obesity (waist-to-hip ratios) should be a simple priority target in intervention programs, since expanding girth is easily recognized. In this study 280 (76.1%) of those with undiagnosed diabetes would have been identified for screening using this measure. Simply reducing abdominal obesity, or better, preventing its development, could and prevent diabetes, NAFLD, cardiovascular and multiple other adverse health events. Policy and practice strategies to increase higher educational achievement should be a priority as more education has been shown to lead to better awareness and adoption of healthy behaviors.[36,37] Some of the successes in achieving weight loss goals in better educated and wealthier populations need to be replicated in larger, less privileged and poorer communities. If we fail to do this, the long-term costs will be prohibitive.
